# Supplementary material for: Efficient protein incorporation and release by a jigsaw-shaped self-assembling peptide hydrogel for injured brain regeneration
Source: Nat Commun. 2021 Nov 19;12:6623. doi: 10.1038/s41467-021-26896-3 (PMC8604910; doi:10.1038/s41467-021-26896-3)
Supplement: Supplementary file 1 — Supplementary Information [file 41467_2021_26896_MOESM1_ESM.pdf]

Supplementary information

Efficient protein incorporation and release by a jigsaw-shaped self-assembling peptide hydrogel for injured brain regeneration

Authors:
Atsuya Yaguchi[1 #], Mio Oshikawa[2, 3 #], Go Watanabe[3, 4], Hirotsugu Hiramatsu[5,6], Noriyuki Uchida[1], Chikako Hara[2, 3], Naoko Kaneko[7], Kazunobu Sawamoto[7,8], Takahiro Muraoka[1, 3]*, and Itsuki Ajioka[2, 3]*

Affiliations:
[1] Department of Applied Chemistry, Graduate School of Engineering, Tokyo University of Agriculture and Technology, Tokyo 184-8588, Japan.
[2] Center for Brain Integration Research (CBIR), Tokyo Medical and Dental University (TMDU), Tokyo 113-8510, Japan.
[3] Kanagawa Institute of Industrial Science and Technology (KISTEC), Kanagawa 243-0435, Japan
[4] Department of Physics, School of Science, Kitasato University, Kanagawa 252-0373, Japan.
[5] Department of Applied Chemistry, National Yang Ming Chiao Tung University, Hsinchu 30010, Taiwan.
[6] Center for Emergent Functional Matter Science, National Yang Ming Chiao Tung University, Hsinchu 30010, Taiwan.
[7] Department of Developmental and Regenerative Neurobiology, Institute of Brain Science, Nagoya City University Graduate School of Medical Sciences, Aichi 467-8601, Japan.
[8] Division of Neural Development and Regeneration, National Institute for Physiological Sciences, Aichi 444-8585, Japan.

#Equal contributors
*Correspondence to: Takahiro Muraoka (muraoka@go.tuat.ac.jp) and Itsuki Ajioka (iajioka.cbir@tmd.ac.jp)

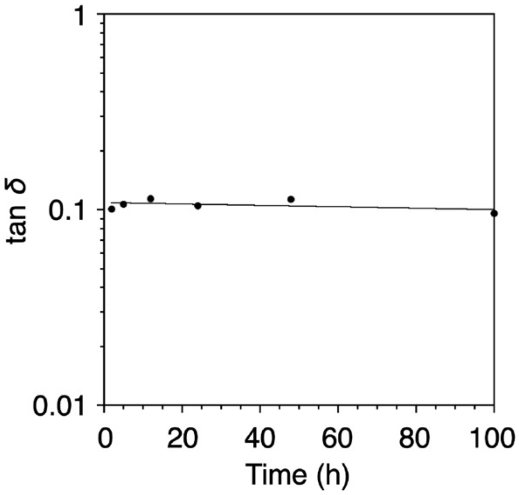

Supplementary Figure 1: Time-course changes in loss factor (tan*δ* = *G*"/*G*') of JigSAP.

JigSAP was dispersed in DMEM containing 1.0 wt% HEPES at 20 °C (peptide concentration: 1.0 wt%, pH 7.4). Source data are provided as a Source Data file.

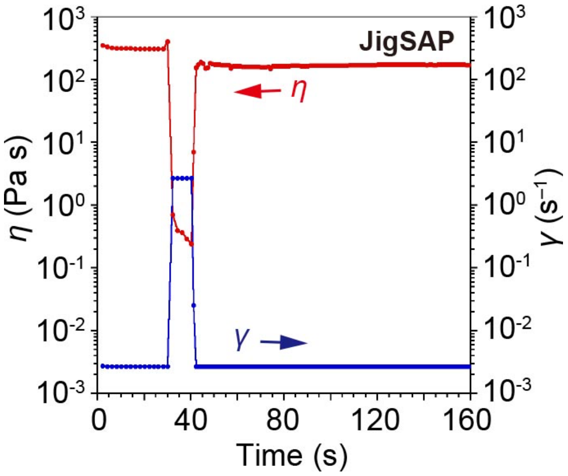

Supplementary Figure 2: Thixotropic properties of JigSAP hydrogel.
Three interval thixotropy tests of hydrogels composed of **JigSAP** at 20 °C, showing changes in viscosity (η, red) at two different applied shear rates (γ, blue) using a step procedure. Source data are provided as a Source Data file.

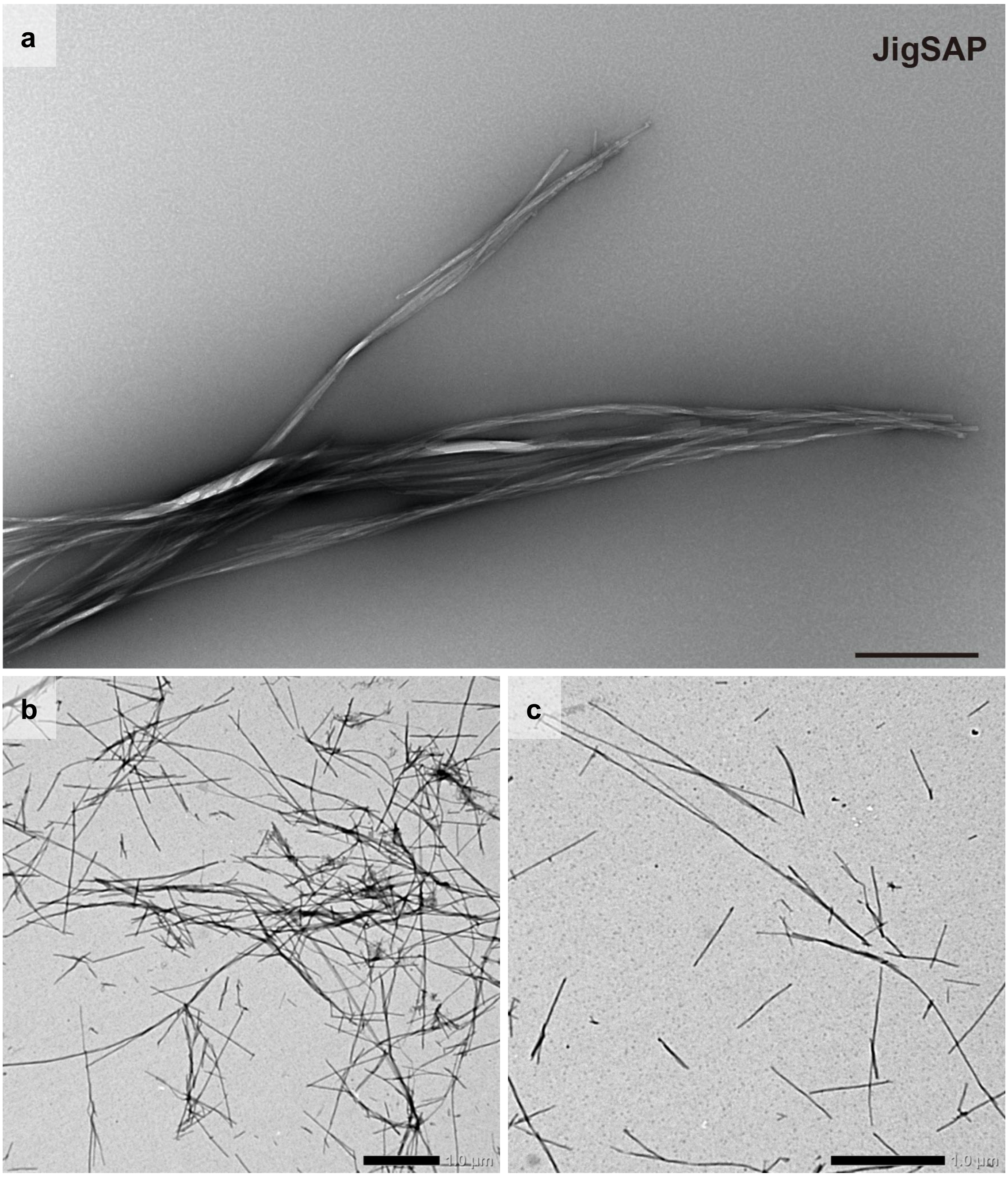

Supplementary Figure 3: TEM image of JigSAP peptide.

After 24 h (**a**, **b**) and 10 s (**c**) aging. Peptide concentration: 0.10 wt%, aging time: 10 s, solvent condition: 0.88 wt% aqueous solution of $NaHCO_3$, uranyl acetate concentration: 2.0 wt%. Scale bars: 400 nm (**a**) and 1 μm (**b**, **c**).

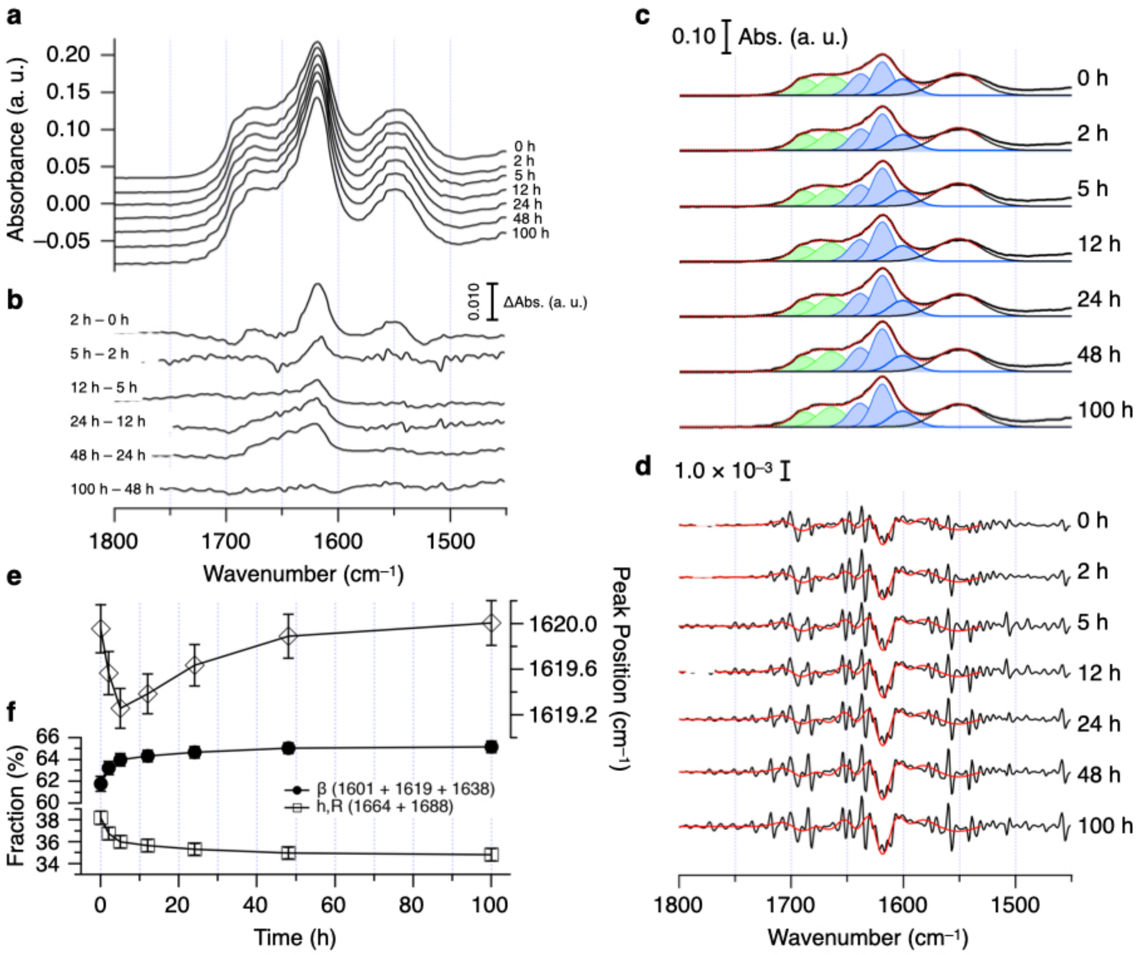

Supplementary Figure 4: IR absorption measurement of JigSAP.

IR absorption measurement of **JigSAP** in DMEM containing 1.0 wt% HEPES at 20 °C at 0, 2, 5, 12, 24, 48, and 100 h after starting incubation (peptide concentration: 1.0 wt%, pH 7.4). **a**, the IR absorption spectrum at each time, and **b**, their difference. **c**, Band decomposition and **d**, second derivative analyses of the IR spectra. Black and red lines indicate the measured IR spectrum and the fitted curve, respectively, in **c**, and the second derivative of the measured IR spectrum and the fitted curve, respectively, in **d**. In **c**, the blue bands are assigned to the β-sheet structures and the green bands are to the others (α-helix and random structures). **e**, Time-course changes of the peak position of the amide I band around 1620 cm^{-1}. n=3. Data are mean ± SEM. **f**, Time-course changes of the fractions of the β-sheet structure (filled circles) and the others (open square) in the amide I band of **JigSAP** evaluated by the deconvolution analysis shown in **c**. n=3. Data are mean ± SEM. Source data are provided as a Source Data file. Abbreviation; a.u.: arbitrary units.

All the IR spectra shown in this study were measured in the hydrated state using CaF_2 windows, of which the path length is fixed (Biocell; Biotools, FL). We performed time-course measurement of IR spectra of **JigSAP** as shown in Supplementary Fig. 3a. Differential spectra indicated a two-step process during the gelation of **JigSAP** (Supplementary Fig. 4b). In the first stage (stage 1: 0–5 h), the differential spectra showed positive peaks at 1677 and 1620 cm^{-1} assigned to the Arg side chains and β-sheet structure, respectively. In the second stage (stage 2: 12–100 h), the differential spectra showed a broad band having the positive peaks at 1620 cm^{-1} corresponding to β-sheet structure.

Band decomposition analysis of the IR spectra of **JigSAP** was carried out by using five Gaussian bands (Supplementary Fig. 4c). The observed IR spectrum at each time (black) was fitted well (red). Also, the second derivative of the fitted curve (red in Supplementary Fig. 4d) reproduced the second derivative IR spectra (black) well. Hence the band decomposition was successful. The bands below and above 1640 cm^{-1} were assigned to the β-sheet structure and the α-helix or random structures, respectively, since the amide I bands of the helical and random structures overlap above 1640 cm^{-1}.

The time-course plot of the amide I peak position showed that the gelation process of **JigSAP** involves two steps (Supplementary Fig. 4e). The band area of each secondary structure was summed and plotted against the time (Supplementary Fig. 4f). The fraction of β-sheet structure increased in stage 1 and reached a plateau after 12 h, while that of the other secondary structures decreased accordingly. The plot did not show remarkable changes in stage 2.

The two-step IR spectral change visualizes the nanofiber formation process of **JigSAP**. Namely, **JigSAP** forms an aggregate, possibly kinetically stable forms, in stage 1. The increased band intensity of the Arg side chains at 1677 cm^{-1} suggests the environmental change of the side chain structure, such as the salt-bridge formation (Supplementary Fig. 4b). This step accompanies the growth of the β-sheet structure because the amide I band at 1620 cm^{-1} becomes larger. Then, the β-sheet structure is likely rearranged into thermodynamically stabler forms to allow for the growth of nanofibers over several-μm in length in stage 2, because the spectral change covered the broad region without changing the fraction of each secondary structure.

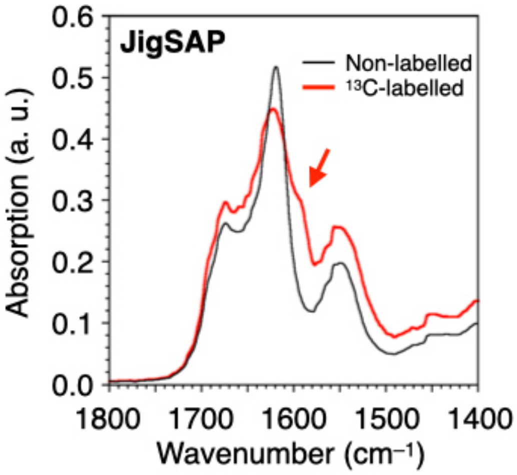

Supplementary Figure 5: IR absorption measurement of JigSAP.
IR absorption spectra of JigSAP (black line) and ^{13}C-labeled **JigSAP** (red line) in DMEM containing 1.0 wt% HEPES after 48 h incubation (peptide concentration: 1.0 wt%, pH 7.4). Arrow points to the absorption band corresponding to ^{13}C=O stretching. Source data are provided as a Source Data file. Abbreviations; a.u.: arbitrary units.

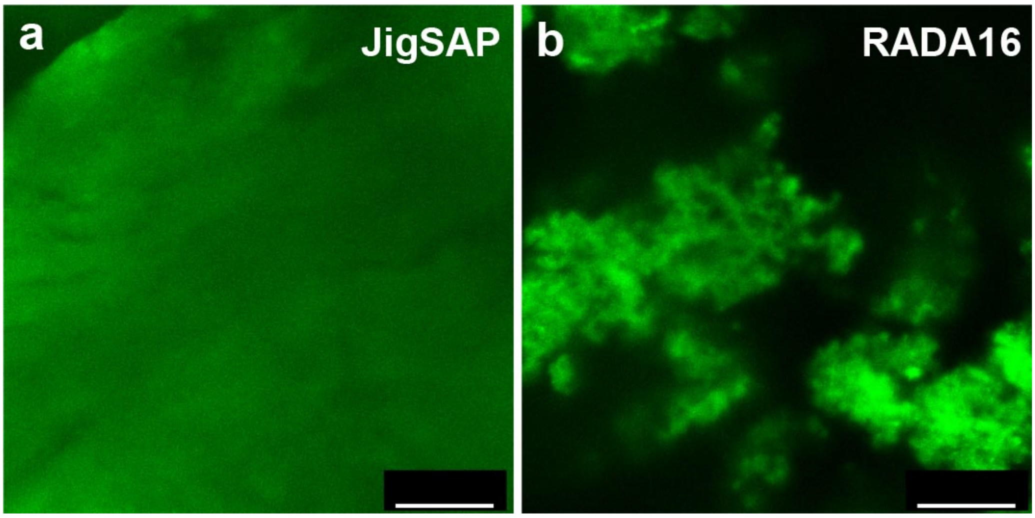

Supplementary Figure 6: Confocal microscopic images.

Confocal microscopic images of fluorescein-labeled **JigSAP** (**a**) and **RADA16** (**b**) hydrogels at 25 °C (peptide concentration: 1.0 wt%). Scale bars: 50 μm.

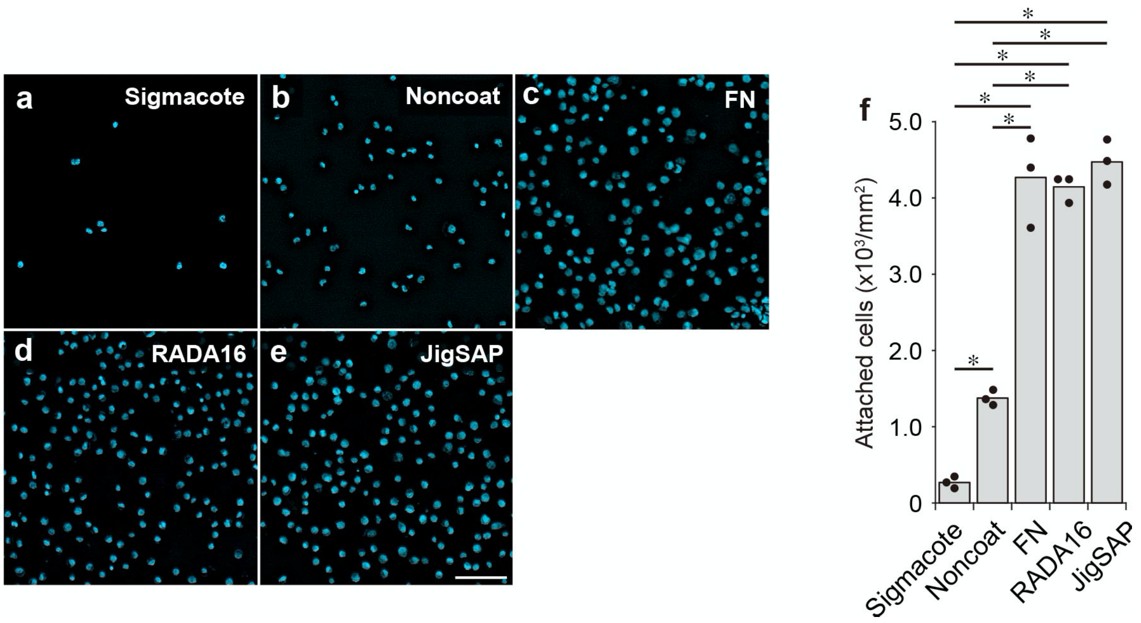

Supplementary Figure 7: Cell adhesion property of JigSAP peptide.
a–**e**, DAPI fluorescent images of fibroblasts attached on a chamber slide coated with Sigmacote (**a**), uncoated control (**b**), Fibronectin (FN) (**c**), **RADA16** (**d**), and **JigSAP** (**e**). Scale bar: 50 µm. **f**, Cell scoring of attached fibroblasts by stereology. *P<0.05 (Student's t-test against Sigmacote and Noncoat, two-sided). Data are mean value. n=3. Source data are provided as a Source Data file.

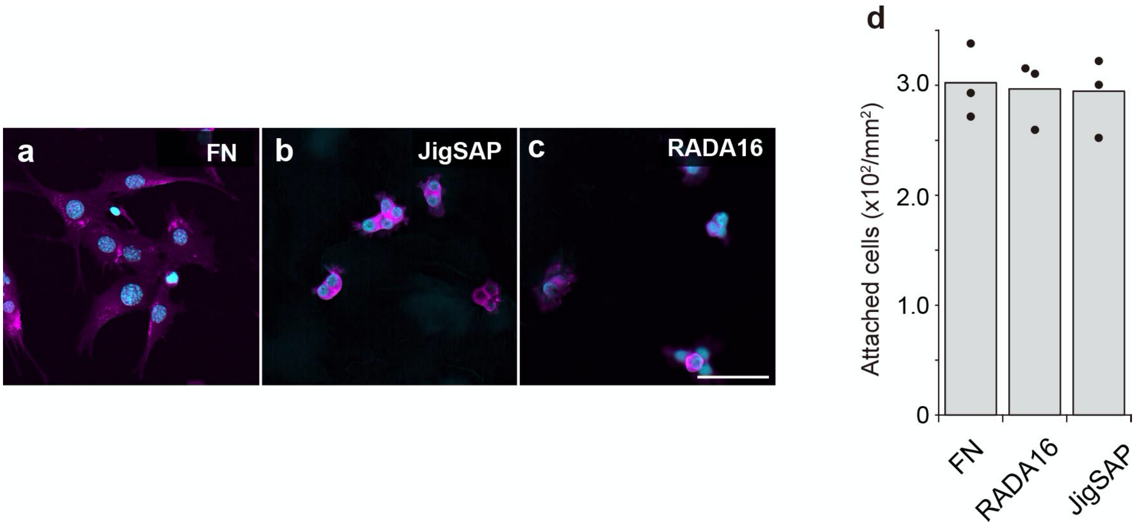

Supplementary Figure 8: Cell morphology cultured on FN, JigSAP, and RADA16. **a**–**c**, Phalloidin (magenta) and DAPI (cyan) fluorescent images of fibroblasts cultured for 24 h on a chamber slide coated with FN (**a**), **JigSAP** (**b**), and **RADA16** (**c**). Scale bar: 50 µm. **d**, Cell scoring of attached fibroblasts by stereology. n=3. Data are mean value. Source data are provided as a Source Data file.

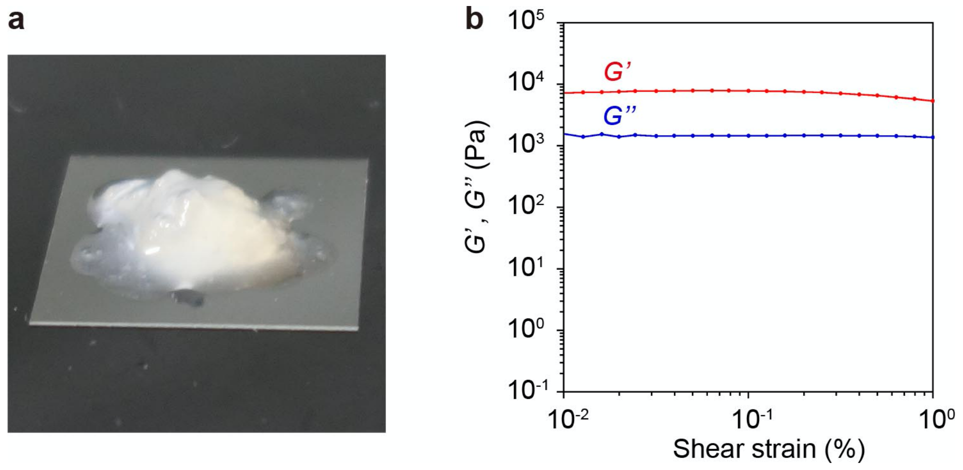

Supplementary Figure 9: JigSAP hydrogel formation in the presence of serum.

a, Photograph of JigSAP hydrogel prepared in DMEM containing 1.0 wt% HEPES and incubated with serum for 48 h. **b**, Strain-dependent storage (*G*', red) and loss (*G*", blue) moduli profile of JigSAP hydrogel. Peptide concentration: 1.0 wt%, buffer volume: 300 µL, serum volume: 2.7 mL. The hydrogel was prepared by 48 h incubation at 37 °C under 5% CO_2 atmosphere. Source data are provided as a Source Data file.

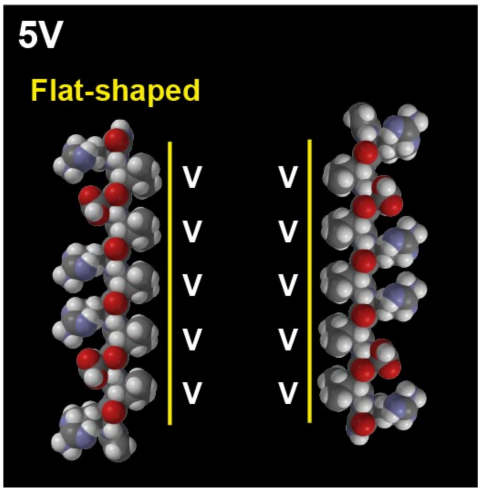

Supplementary Figure 10: Characterization of 5V peptide as a FSAP.
Space-filling models of Ac-RVDVRVRVDVR-NH_2 (**5V**) showing flat hydrophobic surfaces.

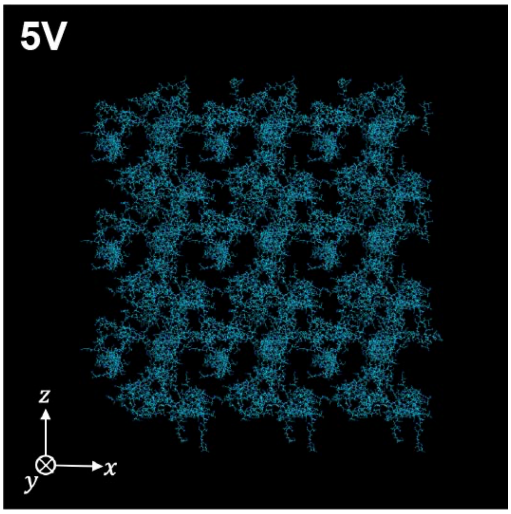

Supplementary Figure 11: Characterization of 5V peptide as a FSAP.
A snapshot of supramolecular structures of **5V** in water at 310 K obtained from an all-atom MD simulation.

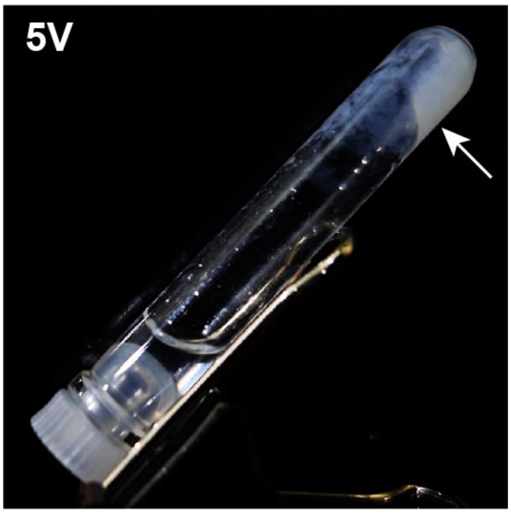

Supplementary Figure 12: Characterization of 5V peptide as a FSAP.
A photograph of **5V** in DMEM containing 1.0 wt% HEPES at 37 °C (peptide concentration: 1.0 wt%, pH 7.4). Scale bar: 5 mm. The arrow and arrowhead point to the viscous suspension and solution, respectively.

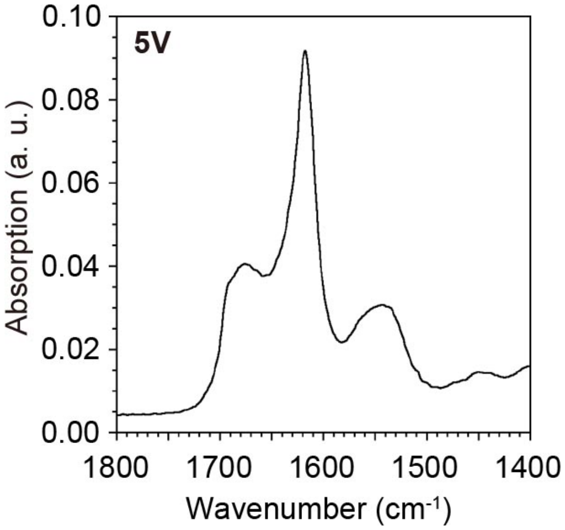

Supplementary Figure 13: Characterization of 5V peptide as a FSAP.
IR absorption spectrum of **5V** in DMEM containing 1.0 wt% HEPES (peptide concentration: 1.0 wt%, pH 7.4). Source data are provided as a Source Data file. Abbreviations; a.u.: arbitrary units.

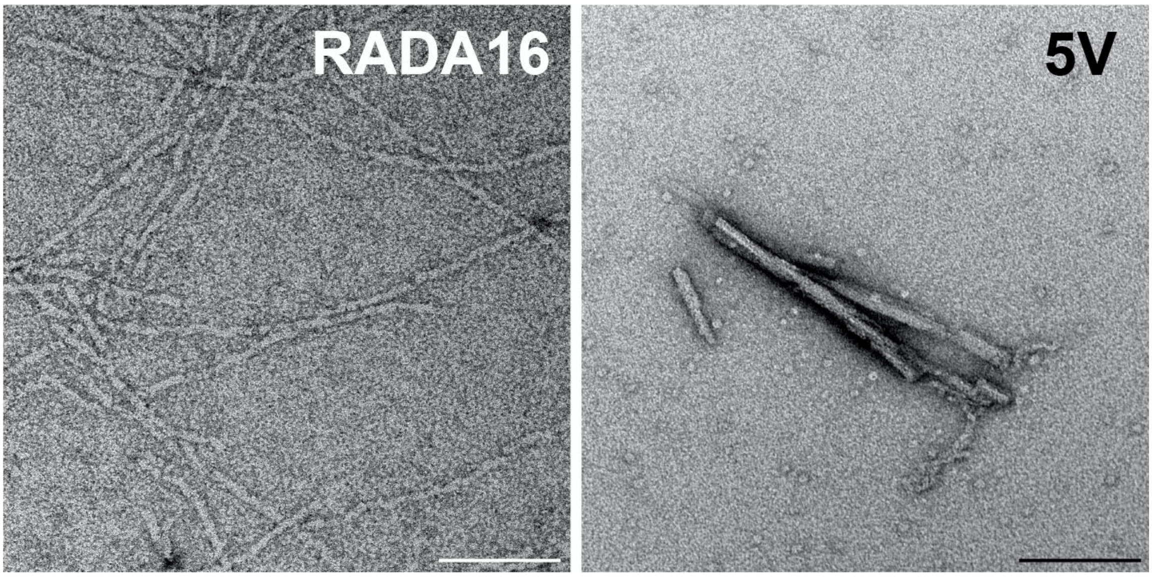

Supplementary Figure 14: TEM images of RADA16 and 5V peptide.
Diameters: 3.9 nm (**RADA16**), 3.3 nm (**5V**), peptide concentration: 0.10 wt%, aging time: 10 s, solvent condition: 0.88 wt% aqueous solution of $NaHCO_3$, uranyl acetate concentration: 2.0 wt%. Scale bars: 100 nm.

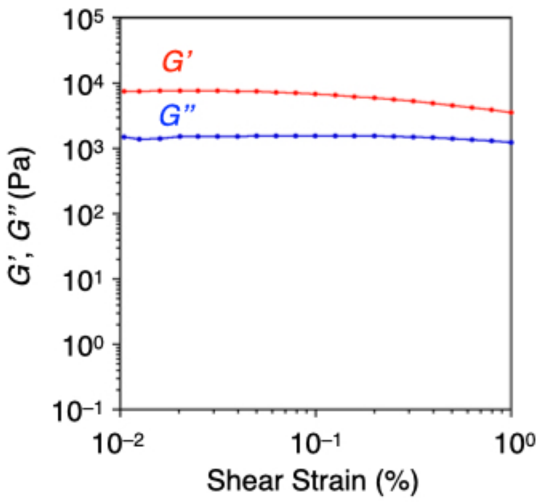

Supplementary Figure 15: Rheology of JigSAP incorporating EGFP-JigSAP. Strain-dependent storage (*G*', red) and loss (*G*", blue) modulus profiles of **JigSAP** incorporating **EGFP-JigSAP** in DMEM containing 1.0 wt% HEPES at 20 °C (incubation time: 48 h, peptide concentration: 1.0 wt%, **EGFP-JigSAP** concentration: 1.0 × 10^{-4} wt%, pH 7.4). Source data are provided as a Source Data file.

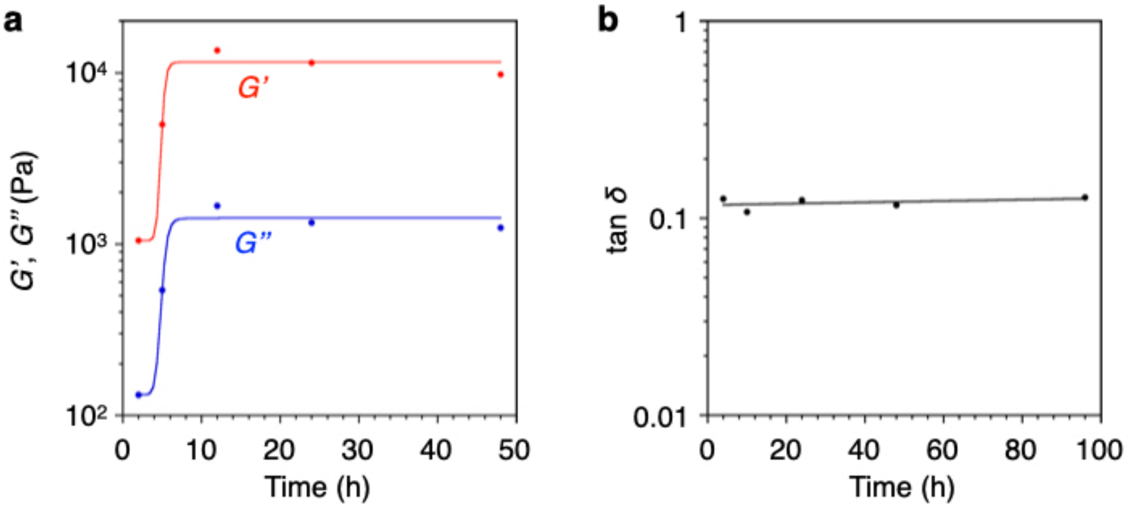

Supplementary Figure 16: Time-course changes EGFP-JigSAP rheology. Time-course changes in (**a**) strain-dependent storage (*G*', red) and loss (*G*", blue) moduli and (**b**) loss factor (tanδ = *G*"/*G*') of **JigSAP** incorporating **EGFP-JigSAP** in DMEM containing 1.0 wt% HEPES at 20 °C (peptide concentration: 1.0 wt%, **EGFP-JigSAP** concentration: 1.0 × 10^{-4} wt%, pH 7.4). Source data are provided as a Source Data file.

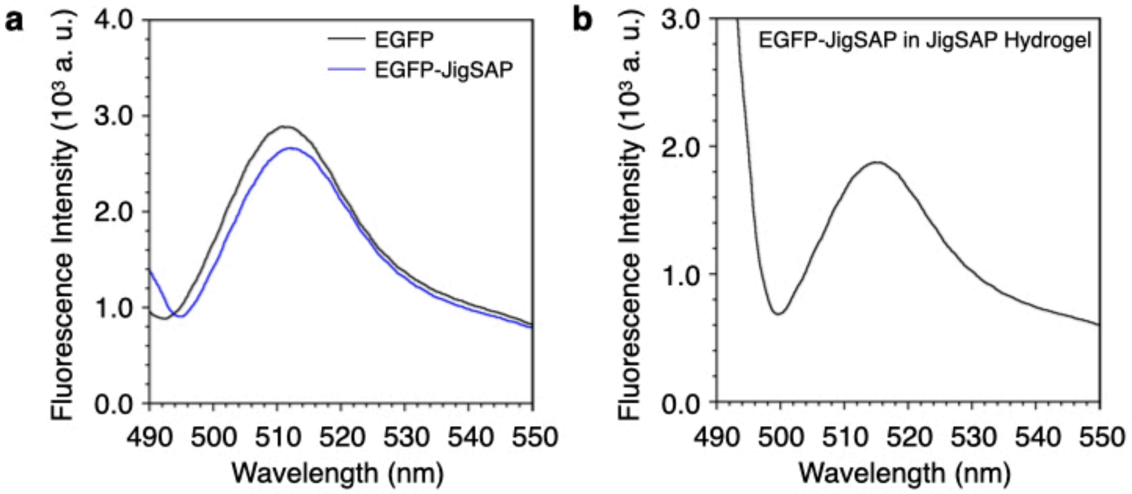

Supplementary Figure 17: Fluorescence spectrum of EGFP-JigSAP incorporated in a JigSAP hydrogel.

Fluorescence spectrum of **EGFP-JigSAP** incorporated in a **JigSAP** hydrogel in DMEM containing 1.0 wt% HEPES at 20 °C (peptide concentration: 1.0 wt%, **EGFP-JigSAP** concentration: 1.0×10^{-2} wt%, pH 7.4, excitation at 480 nm). The **JigSAP** hydrogel incorporating **EGFP-JigSAP** was incubated for 24 h before the measurement. Source data are provided as a Source Data file. Abbreviations; a.u.: arbitrary units.

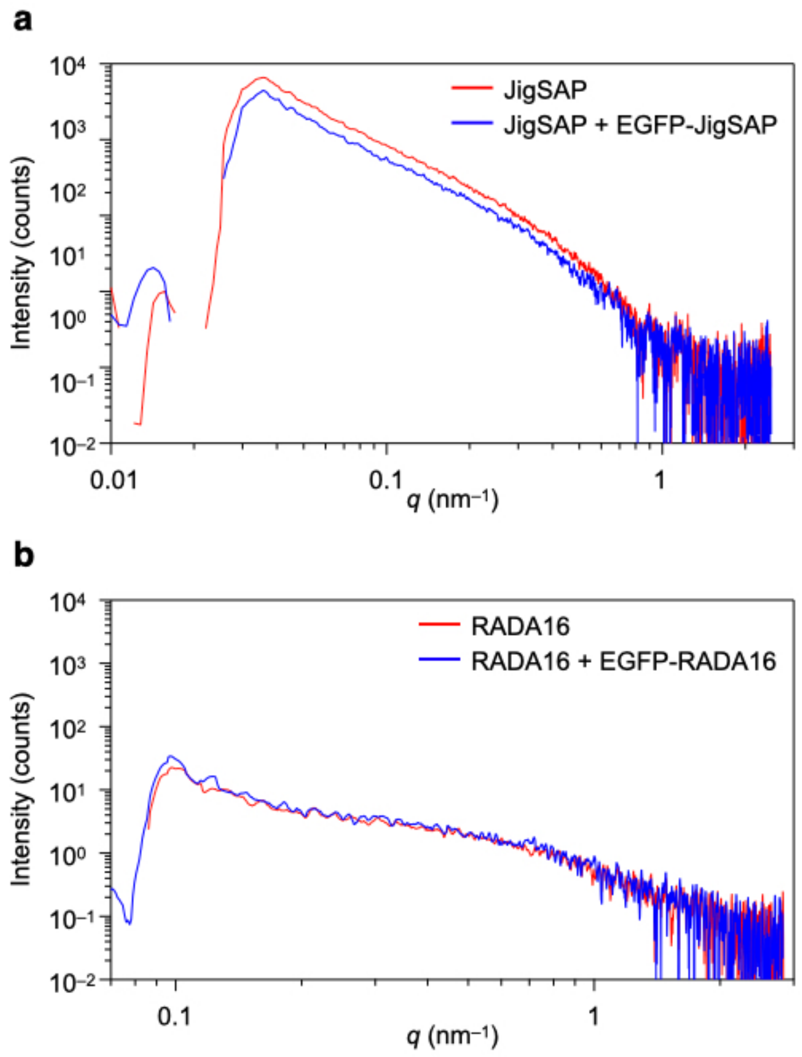

Supplementary Figure 18: SAXS profiles of JigSAP and RADA16 hydrogels. Small-angle X-ray scattering (SAXS) profiles of **a**, **JigSAP** (red line) and **JigSAP** incorporating **EGFP-JigSAP** (blue line) and **b**, **RADA16** (red line) and **RADA16** incorporating **EGFP-RADA16** (blue line) in DMEM containing 1.0 wt% HEPES at 20 °C (peptide concentration: 1.0 wt%, peptide tagged-EGFP concentration: 1.0×10^{-4} wt%, pH 7.4). The **JigSAP** hydrogel incorporating **EGFP-JigSAP** was incubated for 24 h before the measurement. Source data are provided as a Source Data file.

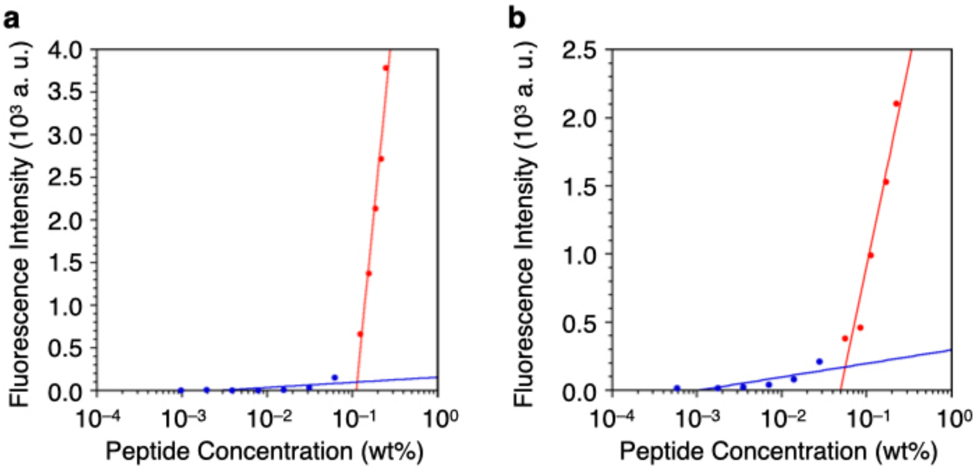

Supplementary Figure 19: Characterization of critical aggregation concentrations (CMCs).

Characterization of critical aggregation concentrations (CACs) of **a**, **JigSAP** and **b**, **5V** in DMEM containing 1.0 wt% HEPES at 25 °C by monitoring ThT fluorescence intensity changes. ThT concentration: 25 μM. Excitation: 440 nm. Fluorescence: 480 nm. The fluorescence spectra were measured with RF-6000 spectrometer of Shimadzu (Kyoto, Japan). Source data are provided as a Source Data file. Abbreviations; a.u.: arbitrary units.

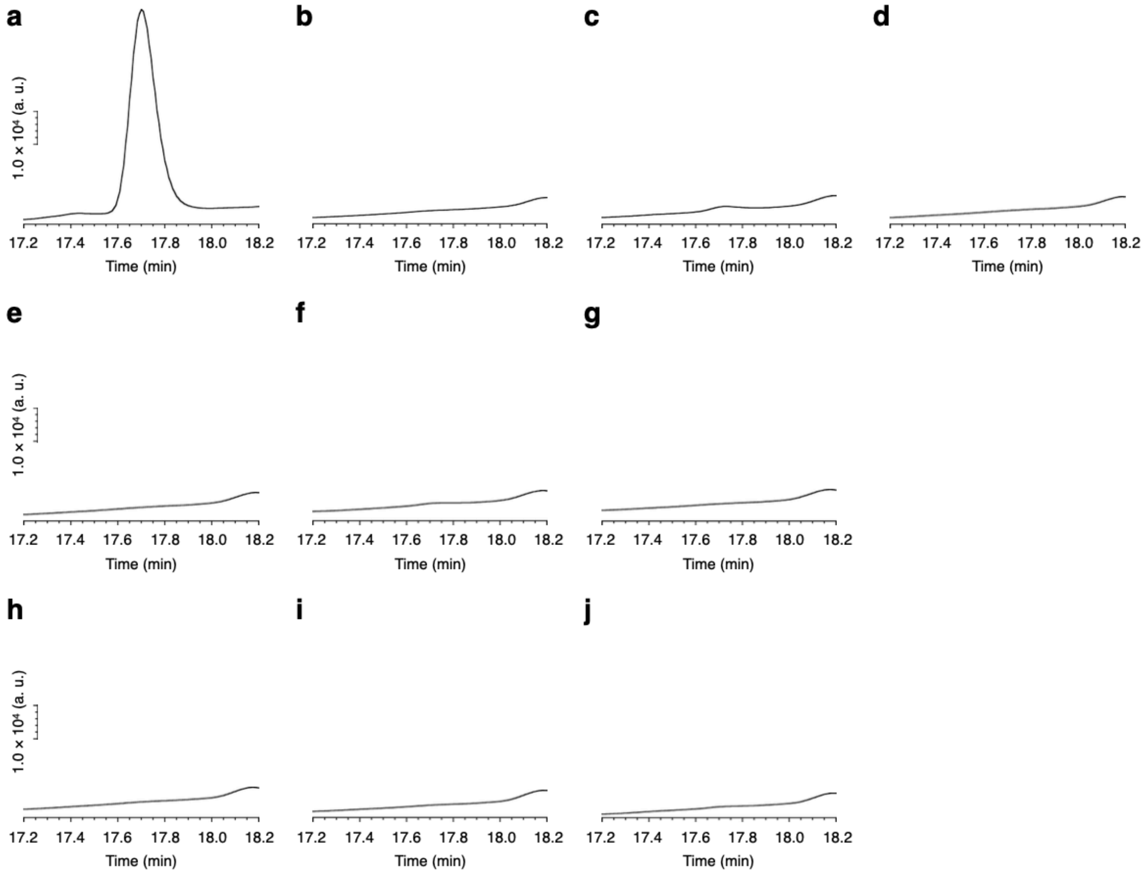

Supplementary Figure 20: No acceleration of hydrogel degradation by protein incorporation.

a–j, HPLC traces of (**a**) **JigSAP** dissolved in buffer (0.010 wt% = 71 μM, not hydrogelated), (**b–d**) supernatants of **JigSAP** hydrogels, (**e–g**) supernatants of hydrogels made of a mixture of **JigSAP** and **VEGF** and (**h–j**) supernatants of hydrogels made of a mixture of **JigSAP** and **VEGF-JigSAP**. Injection volume: 20 μL, Detection by absorption at 220 nm at 25 °C, Flow rate: 1.0 mL min^{-1}, Column: YMC-Triart C18 (TA12S05-2546WT), Apparatus: JASCO UV-4075 and PU-4180 HPLC System, Gradient profile: water/acetonitrile = 90/10 (0 to 5 min), 40/60 (30 min), linear gradient between 5 and 30 min. Source data are provided as a Source Data file. Abbreviations; a.u.: arbitrary units.

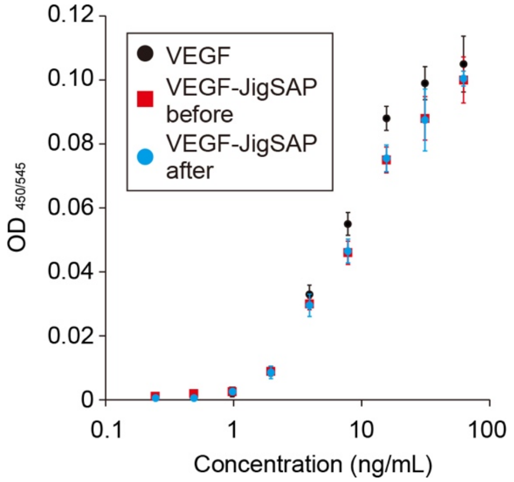

Supplementary Figure 21: Bioactivity of VEGF-JigSAP.

HUVEC proliferation assay with **VEGF-JigSAP** before incorporation into **JigSAP** hydrogel (**VEGF-JigSAP** before), **VEGF-JigSAP** after release from **JigSAP** hydrogel (**VEGF-JigSAP** after), or commercially available recombinant VEGF. The "**VEGF-JigSAP** after" was obtained from the supernatant of sustained-release assay after 7 days. The concentration of "**VEGF-JigSAP** before" and "**VEGF-JigSAP** after" was determined by VEGF ELISA. HUVECs were plated on a collagen-coated 96-well plate (2.5×10^3 cells/well) in 100 μL Medium-199 with 0.1% BSA in the presence of "**VEGF-JigSAP** before," "**VEGF-JigSAP** after," or recombinant mouse VEGF 164 (493-MV; R&D). Two days after plating, BrdU was added, and the cells were cultured for an additional 24 h. BrdU incorporation was quantified using the BrdU Cell Proliferation ELISA Kit (ab126556; Abcam). n=3. Data are mean ± SEM. Source data are provided as a Source Data file.

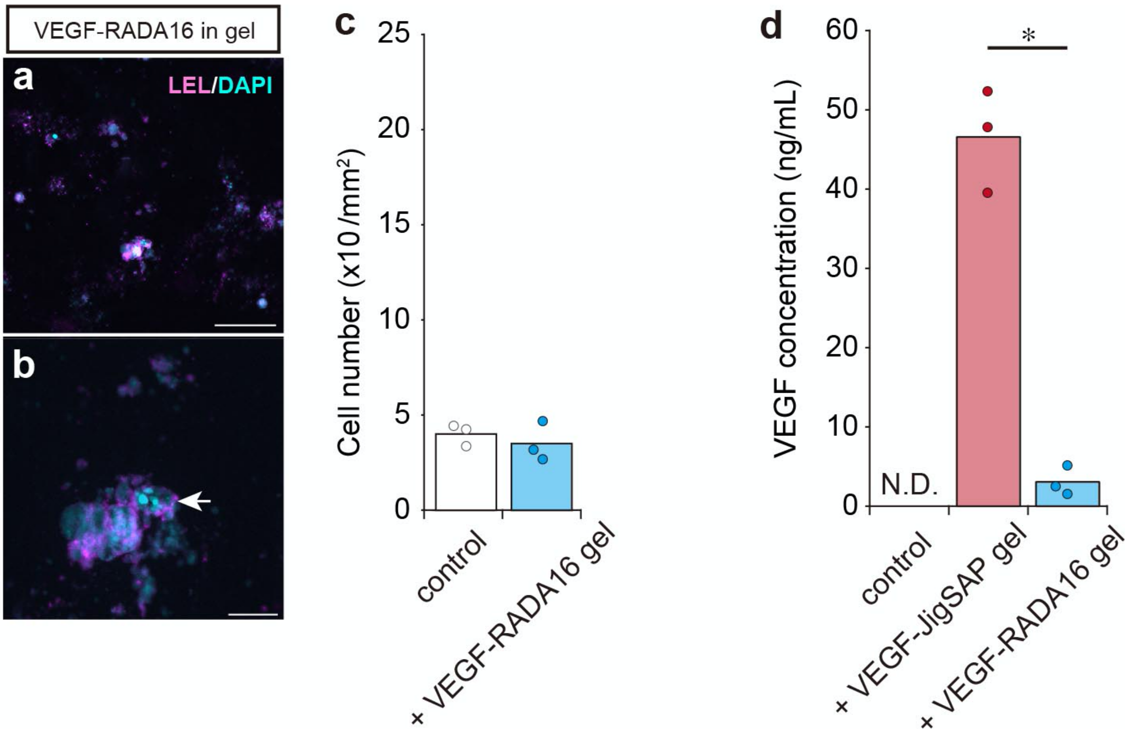

Supplementary Figure 22: HUVEC tube formation assay by VEGF-RADA16.
a, **b**, LEL (magenta) and DAPI (cyan) images of HUVECs. The arrow in (**b**) shows condensed nuclei. Scale bar: 50 µm (**a**) and 20 µm (**b**). **c**, Cell scoring of HUVECs by stereology. n=3. **d**, ELISA assay of the conditioned medium. Data are mean value. n=3. *P<0.05 (Student's t-test against VGEF-RADA16 gel, two-sided). Source data are provided as a Source Data file.

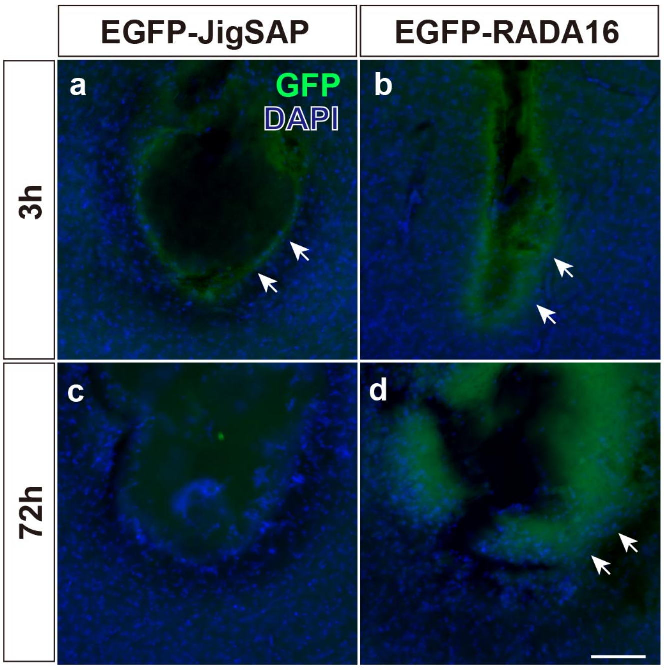

Supplementary Figure 23: EGFP-JigSAP and EGFP-RADA16 injections into the brains.

a-d, EGFP fluorescent images (green) and DAPI (cyan) images of the brains at 3h (**a, b**) and 24 h (**c, d**) after injection. The arrows in (**a, b, d**) show EGFP signal. Scale bar: 50 µm.

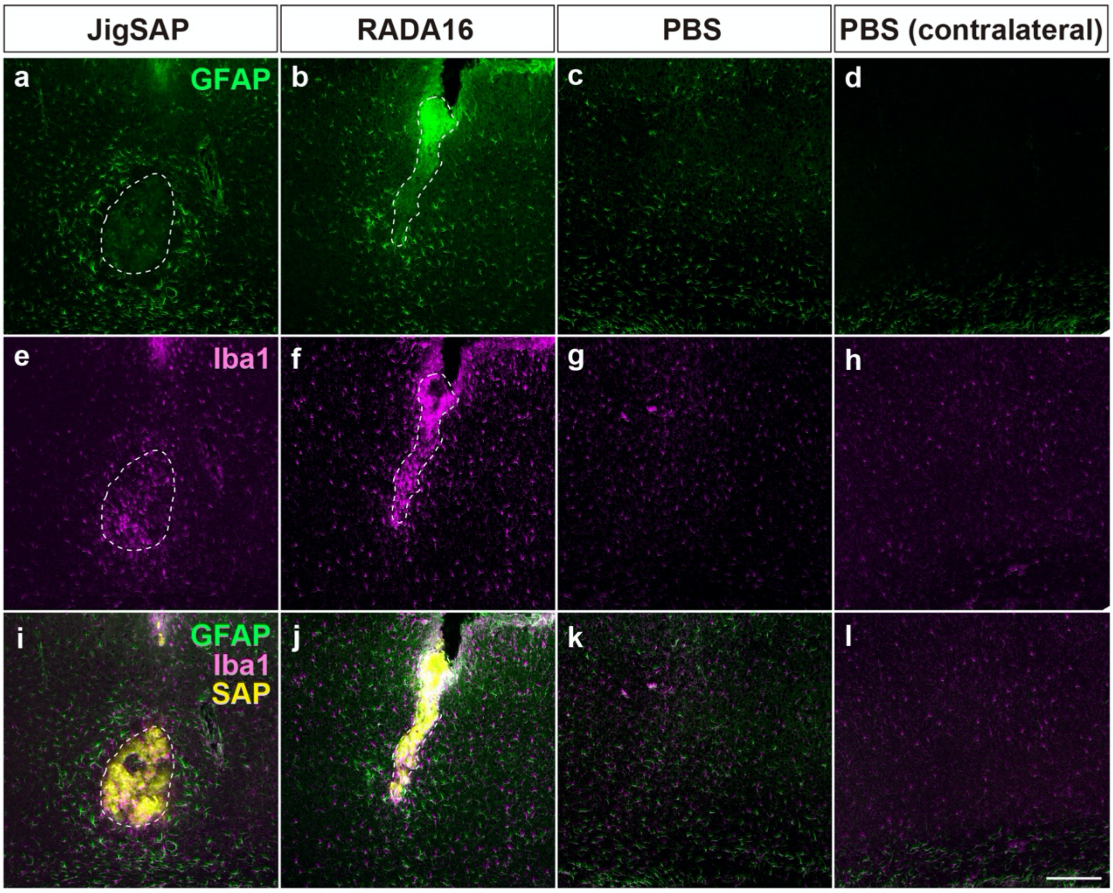

Supplementary Figure 24: Evaluation of the foreign body responses of JigSAP and RADA16 after brain injection.

JigSAP and **RADA16** bearing a 4-pentynoyl group (**Alkyne-JigSAP** and **Alkyne-RADA16**) were used to visualize SAP *in vivo*. 1% **JigSAP** conjugated with 0.01% **Alkyne-JigSAP**, and 1% **RADA16** conjugated with 0.01% **Alkyne-RADA16**, or PBS was injected into the non-injured brain. The injected peptide was visualized by the Huisgen 1,3-dipolar cycloaddition reaction with Alexa 647-azide. At 7 days after injection, these mice were fixed. Activated astrocyte marker GFAP (green) (a-d), microglial marker Iba1 (magenta) (e-h), and merged SAP (yellow) images (i-l). Scale bar: 200 μm.

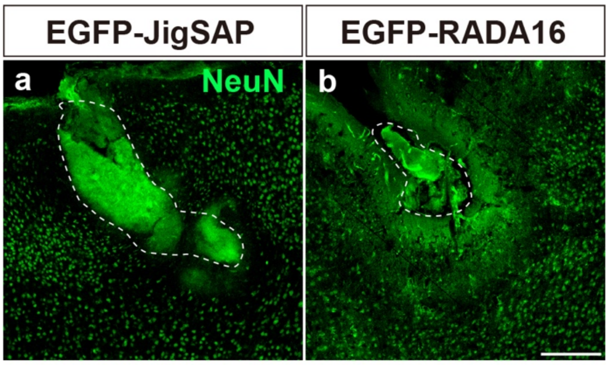

Supplementary Figure 25: NeuN staining after JigSAP and RADA16 injections. **JigSAP** or **RADA16** were injected into the non-injured brain. At 7 d after injection, these mice were fixed. **a, b,** Neuron marker NeuN (green) images. Scale bar: 200 µm.

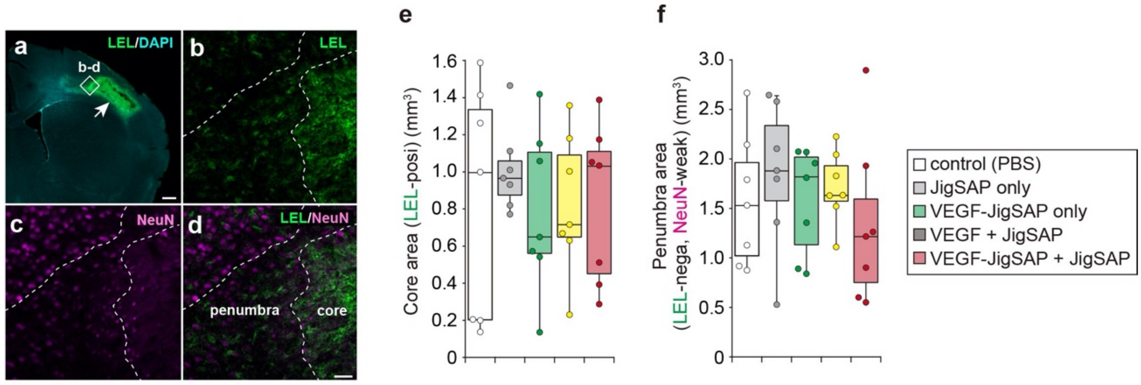

Supplementary Figure 26: Evaluation of the area of the injured core and penumbra after peptide injection.

a–**d**, LEL (green), NeuN (magenta), and DAPI (cyan) images at the injured area of control. LEL stains activated microglia more strongly than endothelial cells. **e**, **f**, The volumes of the core area (**e**) and the penumbra area (**f**) were measured as the LEL-positive region and the LEL-negative and NeuN-weak region, respectively, using the volume measurement function of Stereo Investigator. Scale bars: 500 µm (**a**) and 50 µm (**d**). n=7. Box-plot elements show: center line, median; box limits, 25 and 75 percentiles; whiskers, (Q1/4–1.5IQR) and (Q3/4–1.5IQR). Source data are provided as a Source Data file.

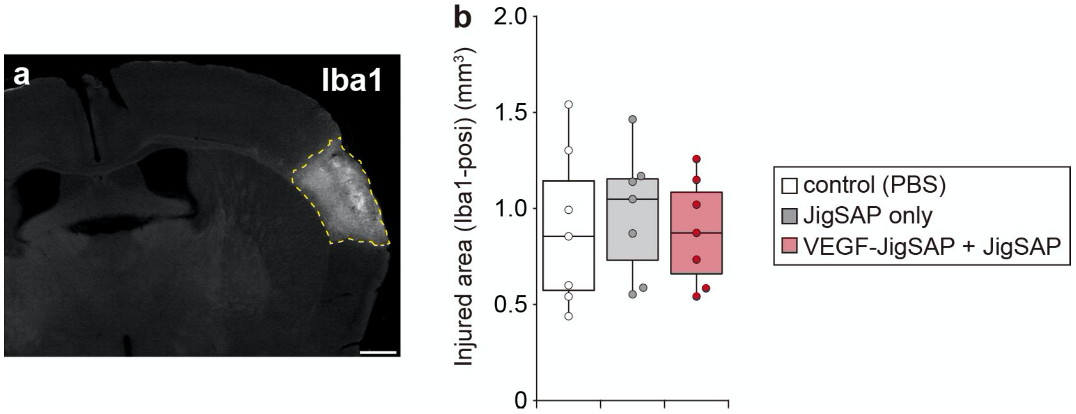

Supplementary Figure 27: Evaluation of the lesion size after peptide injection.
a, Iba1 images at the injured area of control. **b**, The volumes of the Iba1-positive area were measured using the volume measurement function of Stereo Investigator. n=7. Scale bar: 500 µm. Box-plot elements show: center line, median; box limits, 25 and 75 percentiles; whiskers, (Q1/4–1.5IQR) and (Q3/4–1.5IQR). Source data are provided as a Source Data file.

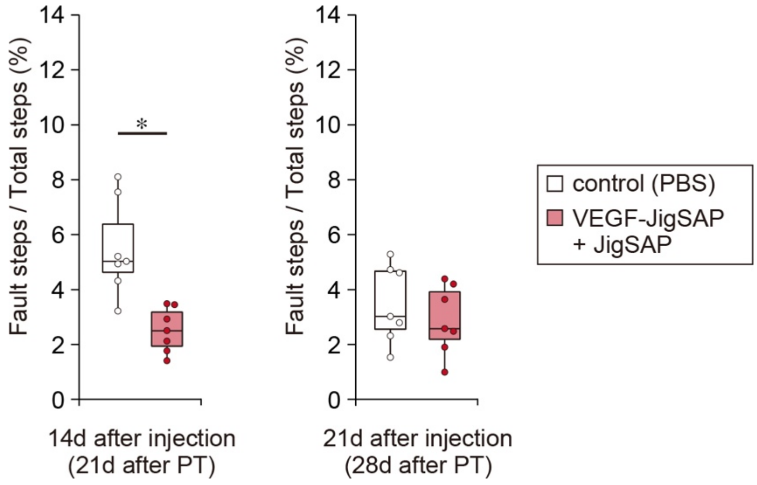

Supplementary Figure 28: Foot-fault test at 14 and 21 days after injection.
Ratios of fault steps to total steps during FFT at 14 and 21 days after injection. *P<0.05 (Student's t-test, two-sided). n=7. Box-plot elements show: center line, median; box limits, 25 and 75 percentiles; whiskers, (Q1/4–1.5IQR) and (Q3/4–1.5IQR). Source data are provided as a Source Data file.

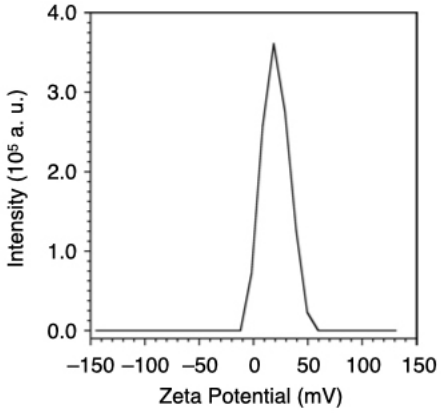

Supplementary Figure 29: Zeta potential profile of JigSAP.

Zeta potential profile of **JigSAP** dispersed at 0.10 wt% in DMEM containing 1.0 wt% HEPES at 20 °C. **JigSAP** in a hydrogel state at 1.0 wt% was dispersed into the buffer for 10-fold dilution immediately before the measurement (peptide concentration: 1.0 wt%, pH 7.4). Source data are provided as a Source Data file. Abbreviations; a.u.: arbitrary units.

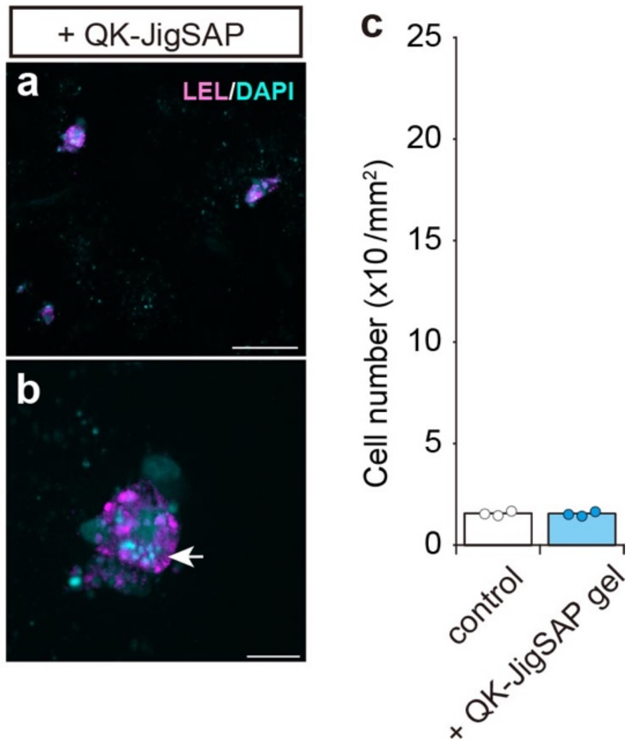

Supplementary Figure 30: QK-JigSAP peptide did not enhance angiogenesis *in vitro*.

a, **b**, LEL (magenta) and DAPI (cyan) images of HUVECs. The arrow in (**b**) shows condensed nuclei. Scale bar: 50 µm (**a**) and 20 µm (**b**). **c**, Cell scoring of HUVECs by stereology. Data are mean value. n=3. **P*<0.05. The y-axis scale was adjusted to that of Figure 3k. Source data are provided as a Source Data file.

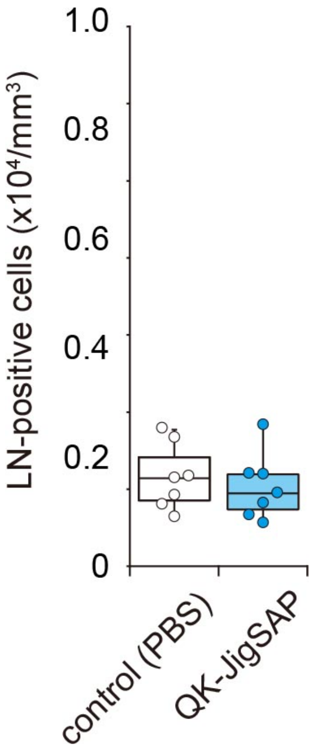

Supplementary Figure 31: QK-JigSAP peptide did not enhance angiogenesis *in vivo*.

Cell scoring of laminin-positive cells at the penumbra. n=7. Box-plot elements show: center line, median; box limits, 25 and 75 percentiles; whiskers, (Q1/4–1.5IQR) and (Q3/4–1.5IQR). The y-axis scale was adjusted to that of Figure 4v. Source data are provided as a Source Data file.

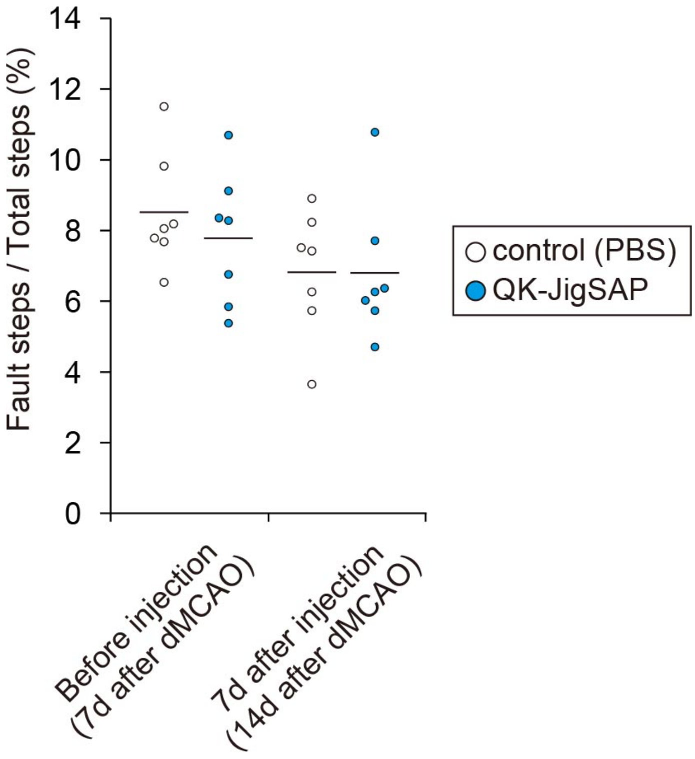

Supplementary Figure 32: QK-JigSAP peptide did not enhance functional recovery.

The ratio of fault steps to total steps during FFT. Data are mean value. n=7. Source data are provided as a Source Data file.

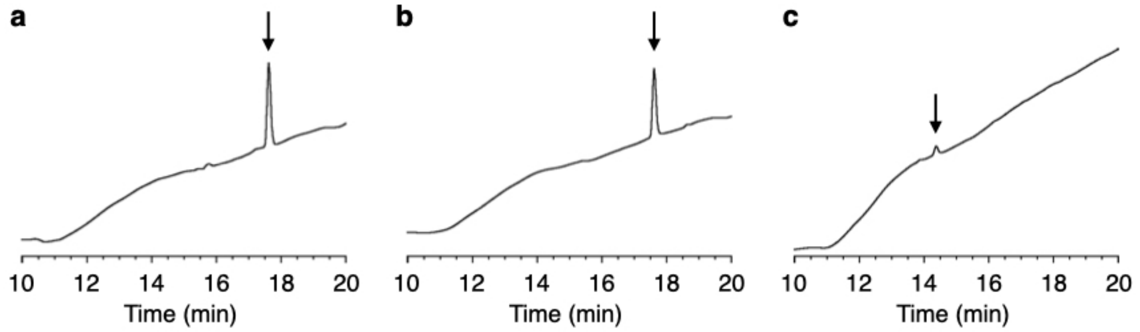

Supplementary Figure 33: HPLC profiles.

HPLC profiles of **a**, **JigSAP**, **b**, **5V**, and **c**, **RADA16** monitored by absorption at 220 nm at 25 °C. The fractions containing each peptide were pointed by arrows. Flow rate: 1.0 mL min^{-1}, Column: YMC-Triart C18 (TA12S05-2546WT), Apparatus: JASCO UV-4075 and PU-4180 HPLC System, Gradient profile: water/acetonitrile = 90/10 (0 to 5 min), 40/60 (30 min), linear gradient between 5 and 30 min. Source data are provided as a Source Data file.

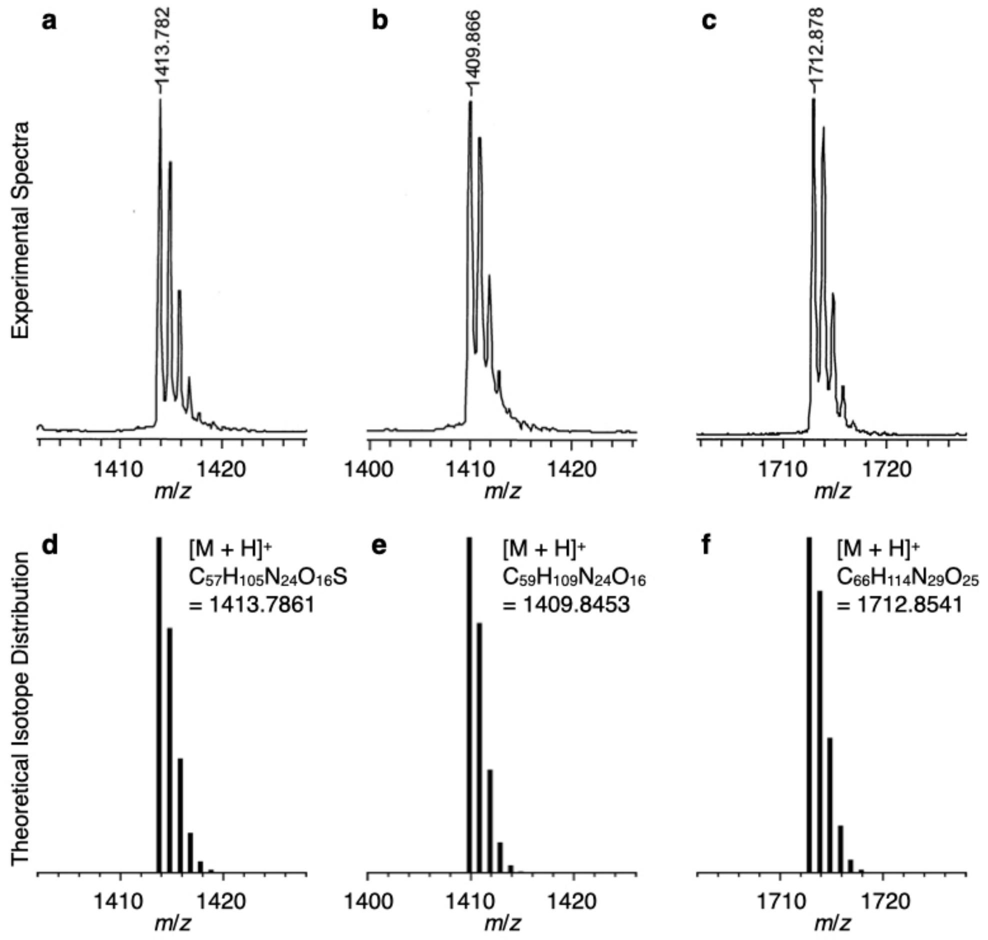

Supplementary Figure 34: MALDI TOF MS data.

MALDI TOF MS data of **a**, **JigSAP**, **b**, **5V**, and **c**, **RADA16** measured by autoflex speed spectrometer of Bruker with a reflector positive mode using 2,5-dihydroxybenzoic acid (DHB) as a matrix. Theoretical isotope distribution profiles of protonated complexes of **d**, **JigSAP**, **e**, **5V**, and **f**, **RADA16** calculated by iMass software (ver. 1.4, Mobile Science Apps).

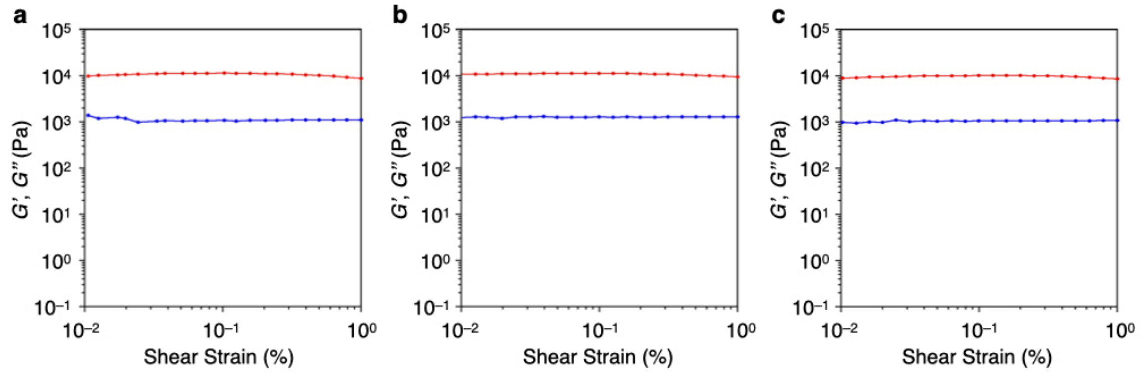

Supplementary Figure 35: strain-dependent storage (*G*', red) and loss (*G*", blue) modulus profiles of JigSAP

Three independent measurements of strain-dependent storage (*G*', red) and loss (*G*", blue) modulus profiles of **JigSAP** in DMEM containing 1.0 wt% HEPES at 20 °C for a replication study (incubation time: 48 h, peptide concentration: 1.0 wt%, pH 7.4). *G*' values at 0.10% shear strain: **a**, 1.14×10^4 Pa, **b**, 1.13×10^4 Pa, **c**, 1.02×10^4 Pa. Source data are provided as a Source Data file.
