# Supplementary material for: Efficient protein incorporation and release by a jigsaw-shaped self-assembling peptide hydrogel for injured brain regeneration
Source: Nat Commun. 2021 Nov 19;12:6623. doi: 10.1038/s41467-021-26896-3 (PMC8604910; doi:10.1038/s41467-021-26896-3)
Supplement: Supplementary file 3 — Description of Additional Supplementary Files [file 41467_2021_26896_MOESM3_ESM.docx]

Description of Additional Supplementary Files

Title: Supplementary Movie 1

Description: A FFT of the non-injured mouse. The non-injured mouse exhibited an accurate limb placement.

Title: Supplementary Movie 2

Description: A FFT of dMCAO mouse. The limb of dMCAO mouse fell down into an opening in the grid.
