# Supplementary material for: Efficient protein incorporation and release by a jigsaw-shaped self-assembling peptide hydrogel for injured brain regeneration
Source: Nat Commun. 2021 Nov 19;12:6623. doi: 10.1038/s41467-021-26896-3 (PMC8604910; doi:10.1038/s41467-021-26896-3)
Supplement: Supplementary file 6 — Reporting Summary [file 41467_2021_26896_MOESM6_ESM.pdf]

nature research

Corresponding author(s): Takahiro Muraoka and Itsuki Ajioka

Last updated by author(s): Oct 18, 2021

Reporting Summary

Nature Research wishes to improve the reproducibility of the work that we publish. This form provides structure for consistency and transparency in reporting. For further information on Nature Research policies, see our Editorial Policies and the Editorial Policy Checklist.

Statistics

For all statistical analyses, confirm that the following items are present in the figure legend, table legend, main text, or Methods section.

n/a	Confirmed	
☐	☒	The exact sample size (n) for each experimental group/condition, given as a discrete number and unit of measurement
☐	☒	A statement on whether measurements were taken from distinct samples or whether the same sample was measured repeatedly
☐	☒	The statistical test(s) used AND whether they are one- or two-sided<br>*Only common tests should be described solely by name; describe more complex techniques in the Methods section.*
☒	☐	A description of all covariates tested
☒	☐	A description of any assumptions or corrections, such as tests of normality and adjustment for multiple comparisons
☐	☒	A full description of the statistical parameters including central tendency (e.g. means) or other basic estimates (e.g. regression coefficient) AND variation (e.g. standard deviation) or associated estimates of uncertainty (e.g. confidence intervals)
☐	☒	For null hypothesis testing, the test statistic (e.g. F, t, r) with confidence intervals, effect sizes, degrees of freedom and P value noted<br>*Give P values as exact values whenever suitable.*
☒	☐	For Bayesian analysis, information on the choice of priors and Markov chain Monte Carlo settings
☒	☐	For hierarchical and complex designs, identification of the appropriate level for tests and full reporting of outcomes
☒	☐	Estimates of effect sizes (e.g. Cohen's d, Pearson's r), indicating how they were calculated

Our web collection on statistics for biologists contains articles on many of the points above.

Software and code

Policy information about availability of computer code

Data collection	GROMACS ver. 2016.6.<br>Stereo Investigator (MBF Bioscience) ver. 10
Data analysis	GROMACS ver. 2016.6.<br>Microsoft Excel (Microsoft) ver. 16.54

For manuscripts utilizing custom algorithms or software that are central to the research but not yet described in published literature, software must be made available to editors and reviewers. We strongly encourage code deposition in a community repository (e.g. GitHub). See the Nature Research guidelines for submitting code & software for further information.

Data

Policy information about availability of data

All manuscripts must include a data availability statement. This statement should provide the following information, where applicable:

- Accession codes, unique identifiers, or web links for publicly available datasets
- A list of figures that have associated raw data
- A description of any restrictions on data availability

Source data are provided as an excel file.
The authors declare that the data supporting this study's findings are available within the paper and its Supplementary Information file.
All other information is available from the corresponding authors upon reasonable request.

Field-specific reporting

Please select the one below that is the best fit for your research. If you are not sure, read the appropriate sections before making your selection.

- [x] Life sciences
- [ ] Behavioural & social sciences
- [ ] Ecological, evolutionary & environmental sciences

For a reference copy of the document with all sections, see nature.com/documents/nr-reporting-summary-flat.pdf

Life sciences study design

All studies must disclose on these points even when the disclosure is negative.

Sample size	No statistical method was used to determine the sample size, and N=7 was used because we could see the significant effects.
Data exclusions	We excluded the sample data from Fig. 4, 5, and 6 and Supplementary Fig. 26, 27, 28, 31, and 32 when dMCAO and photothrombotic models did not produce injured core.
Replication	For animal experiments, we put all of the scores in one graph. All attempts at replication were successful.
Randomization	We did not perform randomization because all experimental animals were purchased from a third company and were already randomized.
Blinding	We kept the movie files of all FFT and performed a blinding test for FFT counting. We also performed a blinding test for cell counting. For other experiments, we did not perform a blinding test because the data were obtained without bias.

Reporting for specific materials, systems and methods

We require information from authors about some types of materials, experimental systems and methods used in many studies. Here, indicate whether each material, system or method listed is relevant to your study. If you are not sure if a list item applies to your research, read the appropriate section before selecting a response.

Materials & experimental systems

n/a	Involved in the study	
[ ]	[x]	Antibodies
[ ]	[x]	Eukaryotic cell lines
[x]	[ ]	Palaeontology and archaeology
[ ]	[x]	Animals and other organisms
[x]	[ ]	Human research participants
[x]	[ ]	Clinical data
[x]	[ ]	Dual use research of concern

Methods

n/a	Involved in the study	
[x]	[ ]	ChIP-seq
[x]	[ ]	Flow cytometry
[x]	[ ]	MRI-based neuroimaging

Antibodies

Antibodies used	chicken anti-GFP antibody (ab19370, Abcam): ELISA 1:5000<br>rabbit anti-GFP antibody (ab290, Abcam): ELISA 1:5000<br>anti-mouse VEGF antibody (AF-493-NA, R&D): ELISA 1:1000<br>biotinylated anti-mouse VEGF (BAF493, R&D): ELISA 1:1000<br>rabbit anti-laminin (ab11575, Abcam): IHC 1:200<br>rabbit anti-Iba1 (019-19741, Wako): IHC 1:2000<br>mouse anti-NeuN (MAB377, Millopore): IHC 1:900
Validation	The company websites show the validation of the primary antibodies.

Eukaryotic cell lines

Policy information about cell lines

Cell line source(s)	HUVEC (obtained rom TaKaRa company)
Authentication	We confirmed a HUVEC character by the response of VEGF.
Mycoplasma contamination	We did not check mycoplasma contamination.
Commonly misidentified lines<br>(See ICLAC register)	No commonly misidentified cell lines were used in the study

Animals and other organisms

Policy information about studies involving animals; ARRIVE guidelines recommended for reporting animal research

Laboratory animals	We used 6-8 weeks C57BL/6J female mice purchased from Charles River Laboratory Japan.<br>All mice were kept in normal light conditions (12:12 light/dark cycles) with unlimited access to food and water.
Wild animals	No wild animals were used in the study.
Field-collected samples	No field collected samples were used in the study.
Ethics oversight	All animal experimental procedures were approved by the Animal Experiment Committee of Tokyo Medical and Dental University (A2021-172C2).

Note that full information on the approval of the study protocol must also be provided in the manuscript.
